# Natural Compounds against Cancer, Inflammation, and Oxidative Stress

**DOI:** 10.1155/2019/9495628

**Published:** 2019-05-09

**Authors:** Claudio Tabolacci, Cinzia Forni, Ravirajsinh N. Jadeja, Francesco Facchiano

**Affiliations:** ^1^Department of Medicine, University Campus Bio-Medico, Rome, Italy; ^2^Department of Oncology and Molecular Medicine, Istituto Superiore di Sanità, Rome, Italy; ^3^Department of Biology, University of Rome “Tor Vergata”, Rome, Italy; ^4^Department of Biochemistry and Molecular Biology, Medical College of Georgia at Augusta University, Augusta, GA, USA

Natural products, compounds derived from animals, micro-organisms and, especially, from plants, have been used in prevention and treatment of various human diseases for thousands of years. Some of these bioactive compounds, which have beneficial effects on human health, are also present in foods and beverages. In recent years, the interest towards the use of natural compounds and their derivatives has been renewed, even for the discovery and development of new drugs [1].

Phytochemicals show positive effects on human health by a large variety of mechanisms, including epigenetic modifications, modulation of signal transduction and metabolic pathways, and regulation of antioxidant enzymes activity [2, 3]. This is the reason why they have been extensively studied for their anticancer activities, through the potential modulation of cancer initiation and growth, cellular differentiation, apoptosis and autophagy, angiogenesis, and metastatic dissemination. Moreover, several active herbal compounds are tested in human clinical trials, used as adjuvants with conventional anticancer therapies in order to reduce side effects like nausea and fatigue [4].

Further, phytochemicals are considered today potent and effective weapons against several human diseases, due to their mechanism of action that in many cases is against oxidative stress. In fact, a considerable number of studies reported the use of natural compounds for their anti-inflammatory activity, since inflammation is considered the basis of various disease conditions, including cancer. The use of herbal preparation in combination with suitable diet represents also one of the most promising strategies to treat metabolic disorders like diabetes, obesity, and metabolic syndrome [5]. Moreover, due to their several biological activities, natural compounds represent good candidates for preventing and even curing the effects of neurodegenerative diseases.

The aim of this Special Issue was to provide a contribution in collecting new findings in the use of natural products against cancer, inflammation, and oxidative stress. After a careful peer review process, twenty-one papers were accepted for publication in this Special Issue, including three review articles and one clinical study.
